# Neural tracking of surprisal in Spanish-English bilingual children during naturalistic heritage language listening^☆^

**DOI:** 10.1016/j.bandl.2026.105833

**Published:** 2026-08-29

**Authors:** Lauren K. Salig, Chi-Lin Yu, Molly Leachman, Nivedhitha Dondati Purushotham, Valeria Ortiz-Villalobos, Zahira Flores-Gaona, Viviana Vélez Negrón, Carla Girardini, Hsin-Yuan Fang, Alisa Baron, Lisa M. Bedore, James R. Booth, Elizabeth D. Peña, Teresa Satterfield, Jonathan R. Brennan, Ioulia Kovelman

**Affiliations:** a The College of New Jersey, Department of Psychology, 2000 Pennington Rd., Ewing, NJ 08628, USA; b University of Michigan, Department of Psychology, 530 Church St, Ann Arbor, MI 48109, USA; c University of North Carolina at Chapel Hill, Department of Psychology and Neuroscience, USA; d Oklahoma State University, Department of Psychology, 403 Psychology Building, Stillwater, OK 74078-3064, USA; e University of Michigan, Department of Computer Science and Engineering, 2260 Hayward Street, Ann Arbor, MI 48109, USA; f University of Rhode Island, Department of Communicative Disorders, 75 Briar Lane, Kingston, RI, USA; g Temple University, Department of Communication Sciences and Disorders, Paley Hall, Philadelphia, PA 19122, USA; h Vanderbilt University, Department of Psychology and Human Development, 230 Appleton Place, Nashville, TN 37203-5721, USA; i University of California, School of Education, 3200 Education Bldg, Irvine, CA 92697, USA; j University of Michigan, Department of Romance Languages & Literatures, 812 E Washington St, Ann Arbor, MI 48104, USA; k University of Michigan, Department of Linguistics, 812 E Washington St, Ann Arbor, MI 48104, USA; l The University of Texas at Dallas, Department of Speech, Language, and Hearing, USA

**Keywords:** Bilingual, fNIRS, Computational, Surprisal, Language development, Naturalistic listening, Heritage language

## Abstract

Our understanding of the neurobiology of language development is imprecise, particularly for bilingual children in their heritage language, since much research focuses on monolingual children or societal majority languages. In this study, we use a novel computational approach to quantifying linguistic predictions with fNIRS neuroimaging to examine bilingual children’s (*N* = 88, ages 7–12) brain activity as they listen to a naturalistic story in their heritage language of Spanish. The children demonstrated successful neural tracking of word predictability in their heritage language, suggesting they can form rich linguistic representations in Spanish. There were some trends for reading and listening comprehension abilities in English and Spanish to modulate neural tracking of predictability in a Spanish story, but no effects were reliable enough to interpret with confidence. Overall, this first use of a predictability-based computational, naturalistic listening comprehension approach with bilingual children demonstrates the feasibility of this approach and its utility for studying language development.

## 1. Introduction

Heritage language bilinguals are individuals who speak both the majority language of their society and a language tied to their family and/or community, known as their heritage language. One challenge of heritage language acquisition is that, although these learners are exposed to two languages early in life—during the most sensitive periods of brain development for language—they often lack opportunities to practice their heritage language in broader contexts or to develop literacy in it. For example, while Spanish is among the most common heritage languages in the United States, many Spanish-English bilinguals grow up in environments where English is the majority language, both academically and in many societal contexts. Consequently, their linguistic experiences differ markedly from those of monolingual speakers in either language. Moreover, much of what is known about the neurobiology of language development is derived from studies of monolinguals using experimental paradigms with limited ecological validity (e.g., sentence judgement tasks). To advance both theoretical understanding of bilingualism and methodological approaches to studying the neurobiology of language development, the present study examines the neurobiology of heritage language development using a naturalistic listening comprehension method.

### 1.1. Bilingual children’s heritage language & brain development

Neurobiological accounts of language development emphasize increasing specialization within the left-lateralized frontotemporal networks. Meta-analytic fMRI evidence shows that, in children, language processing engages bilateral superior and middle temporal gyri (STG, MTG) and the left inferior frontal gyrus (IFG). With age, this activity becomes more left-lateralized, with greater recruitment of the left IFG (Enge et al., 2020).

For bilingual children, this developmental trajectory holds true, with research further suggesting that proficiency and experience in each language shape neural responses to that language. For instance, using an auditory sentence judgement paradigm in English, Baron et al. (2023) and Wagley et al. (2024) found that Spanish-English heritage language bilinguals (ages 7–12) showed robust activation to language in left IFG, MTG, and STG regions. Moreover, older children showed a neural response that was less widely distributed within the left hemisphere (Baron et al., 2023), and there was a relationship between MTG activation and dual-language proficiency (Wagley et al., 2024). These findings align with those observed by Jasinska and Petitto (2013), who examined bilingual children’s neural activation in the societal language (English in Canada) using a sentence judgement task, and found effects of English exposure. There is also some evidence for cross-language influences in neural processing. For example, in a naturalistic listening fMRI study by Leon Guerrero et al. (2024), adolescent bilinguals’ functional connectivity during English listening was correlated with their Spanish cloze reading score.

However, such brain and bilingualism research has two major limitations. First, our understanding of the neurobiology of bilingual language development has largely been shaped by studies examining children’s primary language of academic instruction (e.g., English in the aforementioned examples of Spanish-English heritage language bilinguals) or a formally acquired second language (e.g., English as a second language in China; see Flores-Gaona et al., 2025, for a review). In contrast, heritage languages offer a distinct context for examining how the developing brain facilitates language acquisition under conditions of variable input and unequal language instruction across the two languages. Although the heritage language is typically a child’s first language of acquisition, individual differences in proficiency and use are considerably broader than those observed in monolingual first language development. For example, reductions in heritage language use at home often lead to weaker heritage language proficiency without necessarily producing gains in the academic or societal language, which is primarily supported through schooling and broader social contexts (e.g., Bedore et al., 2016). By focusing on children’s heritage language, the present study aims to advance neurobiological models of language development to encompass a wider continuum of language experiences, particularly those rooted in children’s family and community contexts.

Second, the use of isolated language stimuli, such as sentence judgement tasks (e.g., assessing whether an individual sentence such as “*The box pushed the dog”* is meaningful), has been the primary approach for mapping the brain bases of adult language function as well as developmental shifts (e.g., Skeide et al., 2016; Wang et al., 2024; c.f. story listening tasks with children, e.g., Horowitz-Kraus et al., 2025). Although word- or sentence-level tasks are useful for controlled experiments that isolate key aspects of language processing, they often require metalinguistic judgements and present language in a decontextualized manner that differs from how children use language in their daily lives. Children may engage in language processing differently based on how familiar the task or environment is, and if no contextualizing information is provided, they may make assumptions about context (Satterfield, 2021). Understanding how children capitalize on prior context to efficiently process incoming language input is important to gain a complete understanding of language development and bilingualism (Gu et al., 2025). A clear next step in this work is to determine if prior findings extend to more ecologically valid language processing.

To our knowledge, two studies have investigated bilingual children’s neural processing across both of their languages in naturalistic listening tasks, although neither study specifies if they are evaluating children who speak a heritage language. An electroencephalography (EEG) study by Pérez-Navarro et al. (2024) presented 6-year-old bilingual children in Spain with stories in Basque and Spanish. They found that the children’s neural responses showed stronger acoustic tracking in their less-exposed language and stronger semantic tracking in their more-exposed language. A functional near-infrared spectroscopy (fNIRS) study by Li et al. (2020) presented preschoolers who were second language learners of English in China with auditory stories accompanied by images. They found neural differences in how the children were processing each language and observed that competence in the weaker language (in their case, English) modulated neural responses to that language. The general alignment of these findings with those using more traditional neuroimaging methods supports the validity of the approach. The findings by Pérez-Navarro et al. (2024) are especially encouraging because the children had similar proficiency levels across the two languages, suggesting that this imaging approach can reveal nuanced processing differences in heritage bilinguals.

### 1.2. Neurobiology of language processing using surprisal in naturalistic listening

Recent neuroimaging work with adults takes a computational approach to demonstrate that adults’ brains are sensitive to how surprising each word is in context when processing language during naturalistic listening. In such work, instead of crafting highly controlled stimuli, a corpus of text or, more recently, a pre-trained large language model can be used to calculate the conditional probability of each word in a story (or other natural stimuli) based on the preceding context. The logarithm, to the base 2, of the inverse of this probability is *surprisal*, or amount of information conveyed by the word given the context (Hale, 2016). The surprisal values of each word can be used to predict participants’ neural response in a naturalistic listening context. The key idea behind this approach is that as participants process language, they make predictions about upcoming content because human brains are sensitive to the perceived expectedness/unexpectedness of words within the flow of the sentential context. High surprisal words are less likely to be expected and thus elicit a larger brain response when their arrival conflicts with listener expectations.

Based on prior work, we argue that surprisal serves as a useful metric for capturing broad, multifaceted aspects of language, and more specifically, we expect the neural responses to surprisal in naturalistic language to be a good general indicator of language processing. For example, adults’ neural responses (often measured with fMRI) are sensitive to surprisal or other computationally modelled linguistic features in naturalistic listening of their native language (e.g., Brennan et al., 2020; Gao et al., 2025; Heilbron et al., 2022; Momenian et al., 2024; Willems et al., 2016). One study has taken a surprisal-based approach with children using fNIRS neuroimaging, which is well suited for pediatric populations due to its tolerance for movement and auditory tasks. In that study, Yu et al. (2025) found that monolingual English-speaking children (ages 6–12) demonstrated neural tracking of surprisal in left-lateralized language networks, including IFG, MTG, and STG, with English proficiency modulating the effect. Thus, this approach seems viable for assessing young children’s language processing in a naturalistic context—making it a useful tool to answer our questions about heritage language processing.

Advantages of this surprisal-based analysis approach include its ability to evaluate elements of comprehension that are best studied in naturalistic, contextualized language (e.g., prediction based on prior context), offering different insight than sentence-level tasks, while still revealing word-level sensitivity to language. This approach also moves beyond using neuroimaging to assess overall activation during a task, instead looking for alignment between neural responses and continuous features of language input throughout a task. The use of a naturalistic listening task also does not require metalinguistic judgements or even literacy in the language, making it particularly useful for evaluating processing of a heritage language in which children may have received limited formal instruction.

### 1.3. The current study

Here, we present the first study to use a computational surprisal-based neuroimaging approach to evaluate bilingual children’s heritage language comprehension. In our study, we use fNIRS neuroimaging to evaluate bilingual children’s neural response to surprisal in their heritage language during naturalistic story listening. Our primary aim was to determine if Spanish-English bilingual children in a majority English societal and academic context demonstrate neural tracking of surprisal during naturalistic listening to their heritage language, Spanish. Our secondary aim was to evaluate whether neural tracking of surprisal in the heritage language varies with children’s proficiency in either language, thereby evaluating both within- and cross-language influences.

## 2. Method

### 2.1. Participants

There were 104 Spanish-English bilingual children who completed the study. Of those, 10 datasets were excluded due to technical issues with fNIRS recordings (stimuli mark errors), and 6 were excluded after being identified as outliers (see ‘Preprocessing’ and ‘Analysis’ sections). All tested participants were included unless they failed the neuroimaging-based criteria. Thus, data from 88 participants (49 female, 39 male) were included in the present study’s analyses.

All participants were between the ages of 7 and 12 years *(M* = 9.2, *SD* = 1.3), and we recruited participants who had been exposed to Spanish at home from birth as a heritage language. For the 88 participants, parental report indicated that 84 were Hispanic, 16 were white, 3 were Asian, and 1 was Black (multiple options could be selected). Participants were exposed to English from age 9 or younger (most by age 5 or younger). Participants were recruited in Michigan, a state in the Midwestern region of the United States, where the societal language is predominantly English and only around 3.4% of the population are estimated to speak Spanish at home (U.S. Census Bureau, 2024). Parent reports indicated all students attended English-only schools in the region, although 52% of the children received some Spanish reading instruction. The children received varied amounts of Spanish literacy experience at home as well as with a local Saturday heritage language program. Parents reported the variety(s) of Spanish used by their family, with Mexican Spanish (*n* = 74) being the most represented, although Venezuelan (*n* = 3), Castilian (*n* = 2), Costa Rican (*n* = 2), Colombian (*n* = 1), Cuban (*n* = 1), Nicaraguan (*n* = 1), and Salvadorean (*n* = 1) Spanish were also represented.

During testing, participants completed several behavioral assessments in both Spanish and English (see ‘Procedure’ section). Participant characteristics are shown in Table 1. All participants demonstrated normal nonverbal intelligence (standardized score of 70 or greater on the Test of Nonverbal Intelligence; *M* = 107.73, *SD* = 10.51; Brown et al., 2010), and most participants (*n* = 83) had no parental report of a language or reading disorder. Five participants had a parental report of a language and/or reading disorder; we decided not to exclude those children as they had received intense remediation for years and/or were no longer experiencing language/reading difficulties.^1^

### 2.2. Procedure

Prior to participation, the study procedure (i.e., the tasks involved and the general purpose of better understanding children’s language and literacy) was thoroughly explained to children and their guardians. Children provided assent to participate, and guardians provided consent.

#### 2.2.1. Behavioral assessments

Participants completed a number of behavioral assessments designed to evaluate their Spanish and English abilities, including: the Bilingual English Spanish Assessment Middle Extension (BESA-ME, Peña et al., 2016) to assess morphosyntax skills; the Test of Narrative Language (TNL-2, Gillam & Pearson, 2017; TNL-Spanish Experimental Version, Gillam et al., 2006) in English and Spanish to assess narrative comprehension and production skills; the Test of Word Reading Efficiency (TOWRE-2; Torgesen et al., 2012) in English and the Indicadores Dinámicos del Éxito en la Lectura (IDEL; Plasencia-Peinado et al., 2006) in Spanish to assess word/non-word reading; and sentence reading fluency evaluations in English (subtest of the Woodcock-Johnson IV Tests of Achievement; Schrank et al., 2014) and Spanish (subtest of the Woodcock-Muñoz Test of Cognitive Abilities; Woodcock et al., 2005). Participants also completed the Test of Nonverbal Intelligence (TONI-4; Brown et al., 2010).

Participants’ guardians also filled out a questionnaire about their child’s language background and demographic information, including the Bilingual Input-Output Survey parental survey (BIOS-Home; Peña et al., 2018), which was used to collect estimates of input and output for each language.

#### 2.2.2. Neuroimaging story listening task

Trained lab members explained the neuroimaging procedure to each participant before measuring their head and applying a cap with optodes for the fNIRS recording. Children completed up to three experimental language tasks during their neuroimaging session, the last of which was the Spanish story listening task. Of the 88 participants, 82 completed the fNIRS story listening task in a quiet testing room in our lab and listened to the story played through speakers. The other six participants were tested at a local community center and listened to the story through headphones. For all participants, testers ensured the audio was set at a comfortable and audible volume.

Children were asked to sit as still as possible and look at a fixation cross on screen as they listened to a story in Spanish. The story was adapted from “Juan Bobo busca trabajo” (“Juan goes to work”), a traditional Puerto Rican folktale translated into Spanish by Marisa Montes (2006). Since none of the children tested in this study were acquiring a Puerto Rican variety of Spanish and since the book was published two decades ago, it is unlikely that any of the participants were familiar with this story. For this study, the research team made minor modifications to the story to ensure the vocabulary was understandable for children from different national backgrounds and that the content was age appropriate. For example, the character name Juan Bobo was changed to Juan el Despistado (“Juan, the one who gets distracted easily”). The story was narrated by a Spanish-speaking woman, and character voices were recorded by 5 additional Spanish speakers. The story is 10 min and 8 s long in total, although the first section is not included in the analysis (see ‘Preprocessing’ section), leaving 9 min and 28 s of analyzable fNIRS recording per participant.

At five points throughout the story, the story paused and a pre-recorded voice asked a comprehension question about the story thus far, providing two response options. For example, one question asked which job the main character did particularly well, providing two options: sweeping the floor or putting corn in the basket. Children used a button box to indicate their answer and were provided with immediate feedback on their accuracy before continuing the story listening task.

Participants had high accuracy on the five comprehension questions asked throughout the story (*M* = 0.88, *SD* = 0.18). The majority of participants (*n* = 81) scored 80% or higher, indicating that participants were engaging in the task and understanding basic elements of the story. Two participants scored 20%, three scored 40%, and two scored 60%; however, no participants were excluded for poor task performance based on the idea that language tracking is an automatic process and may be occurring even if children fail to accurately answer questions about the story’s plot.^2^

### 2.3. fNIRS data

Data and analysis scripts are available on OSF (https://osf.io/fxzpk/overview).

#### 2.3.1. Data acquisition

Neuroimaging data was collected using a NIRSport2 system (760 and 850 nm wavelengths) with 12 source and 15 detector optodes, resulting in 32 channels total—17 on the left hemisphere and 15 on the right hemisphere, since one right-side detector was used to create short separation channels (see Supplementary Fig. 1 for the optode array). Optodes were situated in custom-made silicone caps using the international 10–10 system to cover frontal and temporoparietal regions. Trained researchers identified participants’ nasion, inion, and Fpz to ensure consistent cap placement. Prior to beginning the neuroimaging tasks, fNIRS optimization ensured that the signal quality was sufficient and adjustments to the cap and optodes were made as needed.

#### 2.3.2. Preprocessing

Preprocessing of fNIRS data was conducted in MATLAB, using a combination of custom scripts and the NIRS Brain AnalyzIR Toolbox (Santosa et al., 2018). All fNIRS recordings were checked to ensure that stimulus marks were properly recorded; 10 participants with no stimulus marks or incorrect stimulus marks due to system error during recording were excluded. Then, all question and answer segments and the first story listening section (which was used to provide context to generate surprisal regressors in the rest of the story but was not analyzed) were cut out of the recording, leaving the fNIRS recording for the entire analyzable story listening task.

The signal-to-noise ratio for each recording was checked, and one participant whose recording had a signal-to-noise ratio under 5 dB was excluded; this exclusion threshold was set a priori. Next, the original stimulus marks were removed and replaced by stimulus marks indicating the offset of each of the 1153 words (derived using Montreal Forced Aligner; McAuliffe et al., 2017) in the analyzable portion of the story.

During preprocessing, short separation channels (which measure extracerebral signals) were labeled, data was converted to optical density units, TDDR motion correction was applied (Fishburn et al., 2019), data was resampled to 5 Hz, and data was converted to oxygenated hemoglobin (HbO) values by applying the modified Beer-Lambert Law.

#### 2.3.3. Deriving regressors

We included three word-level regressors in our analysis: word rate, log word frequency, and surprisal. Word rate was instantiated as a ‘1’ at the end of each word in the story, and zero otherwise. Log word frequency was calculated by taking the log of the word frequency based on SUBTLEX-ESP (Cuetos et al., 2012) as accessed through CLEARPOND (Marian et al., 2012). Surprisal was our main regressor of interest.

To evaluate language processing, we focused on *surprisal*—a measure of how unexpected or surprising each word is in the context of the story. A similar approach has been used in fMRI and MEG studies with adults, using the brain’s response to word-level surprisal to identify networks of language processing (e.g., Brennan et al., 2020; Heilbron et al., 2022; Willems et al., 2016). Prior work suggests that surprisal correlates with behavioral measures of language processing load in many languages, including Spanish (Wilcox et al., 2023).

We quantified surprisal by submitting the text of the story to GPT2-Spanish (Oñate Latorre & Ortiz Fuentes, 2018), a large, pre-trained artificial neural network that conducts next-word prediction. We used a GPT2 model based on psycholinguistic evidence that models of this size and architecture closely align with human behavior, often more so than much larger models (Oh & Schuler, 2023; Wilcox et al., 2025). This model provided the conditional probability of each word given its preceding context, which was calculated using a 300 s context window. This window was chosen to be as big as possible given the input size of GPT2. For words at the beginning of the story with fewer than 300 s preceding them, all prior context (including the un-analyzed first section of the story) was used to calculate the probability of each word. Surprisal values were generated by taking the negative log probability of each word given that context (Hale, 2001): *surprisal*(Word) = −log_2_(Pr(Word | PriorSententialContext). Surprisal for words consisting of multiple tokens were summed. Word surprisal was correlated with word log frequency (*r* = −0.56, *p* < 0.01); however, our model proportionally splits shared variance, allowing us to assess the linearly independent contribution of surprisal.

### 2.4. Analysis

Participants’ HbO values were analyzed in a subject-level GLM. We used auto-regressive iteratively reweighted least-squares in the model to control for type I errors. This model predicted each participant’s HbO values from the regressors (word rate, log word frequency, and surprisal) by modeling how the brain would respond to each word based on those regressors and a canonical hemodynamic response function with a peak of 6 s (Brauer et al., 2008). That is, the model convolved stick functions at the offset of each word with the hemodynamic response function to generate regressors. Then, five subject-level outliers were identified using the NIRS Brain AnalyzIR Toolbox (Santosa et al., 2018) and were excluded from the subsequent group analysis.

Next, the results of the subject-level GLM were submitted to a group-level linear mixed effects model. This model predicted HbO values based on the regressors (word rate, log word frequency, and surprisal) and included a by-subject intercept as a random effect. Unless otherwise stated, in our results, we report Benjamini-Hochberg FDR-corrected *p*-values (called *q*-values) as calculated by the NIRS Brain AnalyzIR Toolbox (Santosa et al., 2018).

#### 2.4.1. Exploratory ROI analysis

The results of the main analysis showed a localized response to word rate, log word frequency, and surprisal in a specific channel (left-hemisphere channel 7 in Supplementary Fig. 1, corresponding to left STG), which patterned consistently with prior results. Thus, we took the opportunity to conduct an exploratory region of interest (ROI) analysis to investigate how proficiency in each language affects processing of the heritage language.

From the subject-level GLM results, we extracted the model’s per-subject estimate for the effect of surprisal on HbO at this left STG channel. We also extracted the model’s calculated variance for that estimate from the noise covariance matrix. Then, using the brms package (Bürkner, 2021) in R, we ran four Bayesian regression models, one each to assess the relationship of the left STG’s surprisal response with children’s English reading ability, Spanish reading ability, English listening comprehension, and Spanish listening comprehension. Reading ability was operationalized by scores on the sentence reading fluency assessments and listening comprehension by scores on the narrative comprehension assessments (TNL comprehension tasks). Each Bayesian model predicted the left STG surprisal estimates (accounting for the variance of the estimates) from the test score of interest. In each model, age was included as a covariate, since raw test scores were used in the models, and age can have a large impact on scores. In these Bayesian models, we used weakly informative priors: *N*(0,1) for the intercept and *N*(0, 2.5) for other beta coefficients.

## 3. Results

### 3.1. Surprisal predicts left superior temporal brain response

**Fig. 1.**
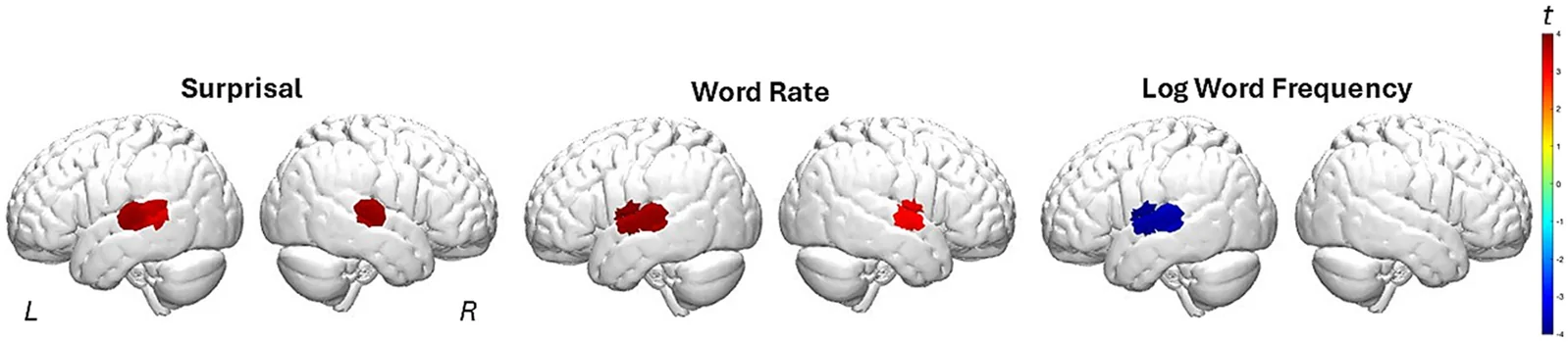
Regressor correlations with neural activity. *Note*. Regressor correlations with recorded HbO values are shown at a *q* < 0.05 threshold, which is corrected for multiple comparisons. Color indicates t-value on a scale of 4 to −4.

The main effects of surprisal, word rate, and log word frequency from the group-level GLM are shown in Fig. 1, with the full model results available in Supplementary Table 1. All three regressors significantly correlated with neural activation in left-hemisphere superior temporal regions. This is in line with prior work on naturalistic listening in fMRI with adults (e.g., Brennan et al., 2016). Word rate corresponded to increased STG activation, while log word frequency corresponded to decreased STG activation. These regressors help confirm that these pediatric recordings show expected neural responses to language: Temporal regions responded strongly to word rate but less strongly for more frequent (and thus easier to process) words.

Importantly, the same STG region also showed a stronger response to more surprising words. This is again consistent with prior fMRI work with adults (Brennan et al., 2016; Willems et al., 2016) and provides evidence that these Spanish-English bilingual children were engaging in successful neural tracking of word expectations in their heritage language.

### 3.2. ROI analysis: brain–proficiency relationships

Our exploratory analyses tested whether the observed effects of surprisal might be modulated by reading or listening proficiency measures in both English and Spanish. This was conducted using Bayesian regression with the dependent measure being the effect of surprisal on the left STG channel which showed a main effect during Spanish naturalistic listening. All four regression models included age as a covariate. Supplementary Fig. 2 shows the median and 95% credibility interval for the estimated correlation between each proficiency measure and the STG effect of surprisal. Supplementary Tables 2–5 show model results. We found a trend for children with greater English listening comprehension ability to show a stronger effect of surprisal in this channel during naturalistic Spanish listening. However, all the coefficients had a 95% credibility interval that included zero, making it difficult to draw any strong conclusions. Model comparison using approximate leave-one-out cross-validation (Vehtari et al., 2017) did not provide evidence to distinguish any one of these models as better than the others.

### 3.3. Exploratory whole brain–proficiency relationships

We also report additional exploratory analyses of whole-brain neural responses to surprisal based on proficiency, as it may be the case that proficiency affects *which* brain regions respond strongly to surprisal. We conducted three additional group-level linear mixed effects models using the results of the subject-level GLM. Each model included main effects of the three regressors (word rate, log word frequency, and surprisal) and a by-subject intercept as a random effect. One model additionally looked for two-way interactions between listening comprehension in English and Spanish (measured with TNL) with the regressors, while another model looked for the same with reading comprehension in English and Spanish (measured with sentence reading fluency). Both of those models included age as a covariate. This allowed us to evaluate how neural tracking of surprisal varied based on our proficiency measures of interest across our entire probeset. Thanks to a reviewer’s suggestion, we also conducted a third model, which included two-way interactions between age and the regressors to directly evaluate how age modulated neural tracking of surprisal in the heritage language. The complete output for these models is available on OSF (https://osf.io/fxzpk/overview).

**Fig. 2.**
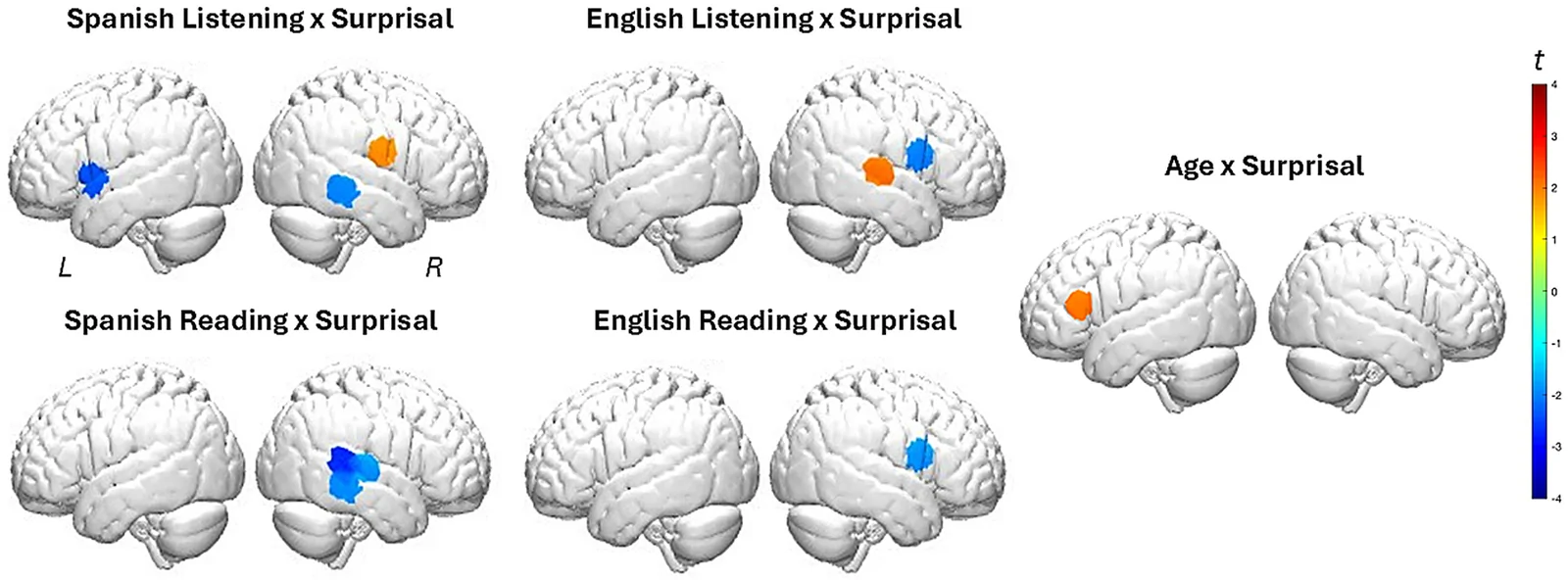
Neural tracking of surprisal by proficiency and age. *Note*. Interactions between the surprisal regressor and proficiency/age measures on the recorded HbO values are shown at a *p* < 0.05 threshold, uncorrected for multiple comparisons; no results survive the multiple comparisons correction. Color indicates t-value on a scale of 4 to −4. English and Spanish listening comprehension were in the same model (age as covariate); English and Spanish reading comprehension were in the same model (age as covariate); and age was in its own model.

As shown in Fig. 2, proficiency in English and Spanish comprehension seemed to mostly affect the involvement of the right hemisphere in tracking surprisal during naturalistic listening. For example, children with greater reading comprehension in either language showed less neural tracking of surprisal in right-hemisphere regions, perhaps suggesting increased left STG specialization as literacy develops. The figure also demonstrates that children with greater Spanish listening comprehension (likely the skill most related to the naturalistic listening task during neuroimaging) showed *less* neural tracking of surprisal in a left frontal region that corresponds to IFG, although this effect is not present when the analysis is re-run with low-accuracy participants excluded. The results also show that older children had a stronger neural response to surprisal in a left IFG region, but the regions that showed a response to surprisal across the whole group (see Fig. 1) did not show any modulation by age.

However, none of the results were significant when corrected for multiple comparisons (*q* < 0.05), again suggesting that we cannot make strong claims about how proficiency or age modulate neural tracking of surprisal based on these data. Regardless, given the novelty of our method, we wanted to be comprehensive in the ways that we explored our data.

### 3.4. Input–proficiency relationship

To better understand how bilingual proficiency was developing in these children, we conducted an exploratory correlation analysis, correlating weekly hours of input in English and Spanish with reading and listening comprehension outcomes (measured with the sentence reading fluency and TNL assessments, respectively). We did not correct for multiple comparisons, as this analysis was exploratory, and we were interested in each input–proficiency relationship as its own theoretical question. As shown in Supplementary Fig. 3, we found that total hours per week of English input did *not* correlate significantly with English reading (*r* = 0.15, *p* = 0.19) or listening comprehension (*r* = 0.20, *p* = 0.08) but did have a weak-to-moderate negative correlation with Spanish listening comprehension (*r* = −0.31, *p* = 0.01). Conversely, total hours per week of Spanish input did not correlate with English reading (*r* = −0.05, *p* = 0.89) or listening comprehension (*r* = −0.14, *p* = 0.80) but did have weak positive correlations with Spanish listening (*r* = 0.25, *p* = 0.01) and reading comprehension (*r* = 0.28, *p* < 0.01).

## 4. Discussion

Using a naturalistic listening neuroimaging approach, this study aimed to advance our understanding of language development and bilingualism by examining the heritage language of young bilingual speakers. Our results indicate that these bilingual children demonstrated successful neural tracking of surprisal during naturalistic listening of their heritage language. This novel finding suggests that young bilingual children with variable and limited exposure to their heritage language develop sensitivity to word-level surprisal in their heritage language—showing neural responses to surprisal in similar regions as those shown in English-speaking adults in response to English listening (e.g., Willems et al., 2016). We can infer that these children have become sensitive to the linguistic probabilities of Spanish and can form rich lexical representations of Spanish sentences and stories, allowing them to be surprised when unlikely words occur. This provides an optimistic outlook for children acquiring a heritage language in a majority English environment and aligns with prior research indicating that there can be meaningful neural responses to language input even for listeners with limited exposure to that language (e.g., Bice & Kroll, 2019).

Notably, though, the children tested in this study were reported to receive substantial amounts of Spanish input (*M* = 46 h per week), despite living in a largely English-speaking societal context. Indeed, it may be the case that our participant recruitment attracted families who were more invested in their children’s bilingualism and who provided more heritage language input, leading to a sample that may not be representative of the average Spanish heritage language bilingual child in a Midwestern United States context. Research conducted within similar contexts has shown that families find it difficult to maintain high levels of heritage language exposure as children grow older and spend more time in English-centric spaces (Rosa, 2019; Vélez-Negrón et al., 2025). Thus, it may well be the case that our sample’s successful neural tracking of heritage language surprisal was dependent on dedicated exposure to and instruction in their heritage language. Factors such as age, heritage language exposure, and individual attitudes toward one’s languages (e.g., Surrain, 2021) may modulate the development of one’s heritage language, requiring further work on the topic to better understand these complex dynamics.

More broadly, our results are in line with prior work showing that left-localized temporal regions are critical for children’s language processing (e.g., Enge et al., 2020) and extend this conclusion to language processing, as reflected by lexical predictions, in more ecologically valid tasks and in bilinguals’ heritage language.

### 4.1. The role of STG in tracking surprisal

Neurobiological perspectives on bilingualism highlight functional and anatomical changes resulting from dual-language experiences during early childhood (Ortiz-Villalobos et al., 2023). Meta-analyses of neuroimaging research with bilinguals place STG at the center of the bilingual brain’s functional plasticity by suggesting that earlier exposed bilinguals more strongly or more focally engage left temporal regions in language processing (Cargnelutti et al., 2019). For instance, Jasinska and Petitto (2013) found different patterns of STG activation in early-relative to later-exposed bilinguals, across both child and adult participants who completed a sentence judgement task. In the context of traditional word- or sentence-level experimental manipulations, these and similar prior works have associated bilingual plasticity in STG functionality with phonological and morphosyntactic processes, such as a larger number of language representations and dual-language co-activation even when only one language is in use, resulting in different language processes in bilinguals relative to monolinguals.

The present work contributes to this discussion by highlighting the role of STG, including bilateral middle STG and posterior left STG, in the context of making word-level predictions about forthcoming language. This STG-localized surprisal effect in bilingual children differs slightly from findings in the most relevant prior work with monolingual children (Yu et al., 2025). While Yu and colleagues report STG as sensitive to surprisal, they found a more widespread neural response to surprisal for English-speaking monolingual children in naturalistic listening—including STG, MTG, IFG, and temporoparietal junctions. Similarly, neuroimaging studies of adults during naturalistic listening generally indicate STG as important for surprisal tracking along with other frontal, temporal, and parietal regions as well (Brennan et al., 2020; Heilbron et al., 2022; Willems et al., 2016). The difference between our surprisal result and Yu and colleagues’ surprisal result may reflect typical variation (perhaps due to sensitivity or task effects) or meaningful differences between bilingual and monolingual or between heritage language and academic language processing. Future research is needed to better evaluate how neural tracking of surprisal varies based on factors such as age, language background, and prior experience with the specific language. Studies such as our own and Yu and colleagues’ provide an example of how a naturalistic listening approach with neuroimaging can address such questions. However, our results only speak to how children respond to lexical surprisal in their heritage language, so future work may also modify this approach to evaluate phonological or morphological elements of language processing.

### 4.2. Proficiency and surprisal tracking

Our secondary aim was to determine if neural tracking of surprisal in the heritage language varied based on proficiency in either language. We started by analyzing the left STG region of interest identified by our main model as particularly sensitive to language. Although there was no reliable effect of any proficiency measure on surprisal tracking in our region of interest, there was a trend toward a stronger response to surprisal in the Spanish story for children with greater English listening comprehension (see Supplementary Fig. 2). This trend could suggest that the children who are able to form strong, word-level expectations in Spanish also become better at extracting information from English narratives, as is required for the English listening comprehension task. Such an explanation would be in line with work showing that early Spanish skills can support later English skills (Marchman et al., 2020). Alternatively, it may be the case that academic instruction in a language supports lexical prediction during listening comprehension across both languages, as would be consistent with predictions on bi-directional bilingual transfer and the views of bilingualism as a singular neurocognitive language system with subsystem differentiation (Marks et al., 2022).

Our whole-brain analysis investigating the role of proficiency in neural tracking of surprisal also revealed some interesting trends but no reliable results. Within the left hemisphere, we found that better Spanish listening comprehension abilities aligned with less neural tracking of surprisal in a frontal region corresponding to left IFG (although this finding was not present when the analysis was re-run without low-accuracy participants). If such a pattern emerges again in future work, it could suggest that children with lower Spanish listening abilities may recruit more regions for neural tracking of surprisal, while children with better Spanish listening abilities may have localized their neural tracking of surprisal more strictly to STG, as indicated by the main effect of surprisal in the main model. We also observed a trend in which older children had stronger neural tracking of surprisal in a left IFG region, which is in line with surprisal tracking results from monolinguals in Yu and colleagues’ study (Yu et al., 2025) and perhaps also with general findings of increased left lateralization for language comprehension with age (e.g., Enge et al., 2020). Spanish listening comprehension and age had directionally different effects on neural tracking of surprisal in left IFG (albeit in slightly different regions). Given that neither result survived the multiple comparisons correction, we cannot meaningfully interpret these results; however, we note that age and heritage language listening comprehension were not correlated in our sample (see Supplementary Fig. 3) and may reflect different sources of variation that future work could explore. Finally, we also found trends such that listening and reading proficiency modulated neural tracking of surprisal in temporal and frontal regions of the right hemisphere. For instance, greater reading ability in either language aligned with less right-hemisphere tracking of heritage language surprisal. This trend suggests that literacy in either language may result in more left-localized and perhaps more efficient tracking of the heritage language.

Despite the lack of reliable brain–proficiency relationships, these findings suggest that further investigation of proficiency, age, or exposure effects may be fruitful, as expected based on prior work and theoretical understandings of bilingualism (e.g., Li et al., 2020; Pérez-Navarro et al., 2024). Future works that include children with more balanced dual-language instruction, as well as neuroimaging data across both languages, are needed to offer a more definitive answer to questions about how language proficiency and background affect bilinguals’ neural processing of language.

#### 4.2.1. Input–proficiency relationship

Although proficiency did not show strong effects on neural tracking of surprisal, our exploratory correlations indicate that input may be an important factor in heritage language proficiency. English input did not correlate with English proficiency measures but negatively correlated with Spanish listening comprehension, while Spanish input supported Spanish proficiency without affecting English proficiency. Although these correlations were weak, this pattern aligns with past work (e.g., Bedore et al., 2016) and suggests that persistent and sufficient exposure to the heritage language is critical for heritage language development without any influence, positive or negative, on children’s primary language of instruction.

It is clear that heritage-language input is important for language development, and that these children were overall able to track surprisal in their heritage language. Although we did not find any reliable relationship between English or Spanish language comprehension ability and neural tracking of Spanish surprisal, we hope future research will continue to look for this type of informative brain–behavior correlation, using our novel naturalistic listening approach as a foundation to build our understanding of heritage language development.

### 4.3. Neuroimaging with naturalistic listening for understanding bilingual development

This study represents the first use of a surprisal-based language comprehension analysis on naturalistic listening comprehension in bilingual children. While many studies have applied a similar approach in fMRI with adults (e.g., Willems et al., 2016), only one study has used this approach in children, also using fNIRS neuroimaging (Yu et al., 2025). Our study demonstrates the feasibility of taking this approach with bilingual children and with languages other than English. This naturalistic listening approach will be particularly useful for studying neural sensitivity to a heritage language, as children often have limited formal instruction or literacy in their heritage language (although this is not necessarily the case for our sample). Further, the naturalistic listening approach more closely parallels children’s day-to-day language exposure, as compared to isolated word- or sentence-level tasks, and does not require any metalinguistic judgements—improving alignment between lab-based tasks and real-world language use. Overall, this study provides a blueprint for how to use a surprisal-based neuroimaging approach with bilingual children, providing benchmarks of how to evaluate data quality (e.g., sensible effects of word rate and word frequency), how to interpret results, and how to investigate the effect of proficiency on neural responses.

### 4.4. Conclusions

Using fNIRS neuroimaging, we found that bilingual children in a majority English context demonstrated neural sensitivity to word-level surprisal in their heritage language, Spanish. Although neither English nor Spanish proficiency reliably predicted the neural effect of surprisal in the region most sensitive to language effects, exploratory correlations suggested that maintaining heritage language input is important for heritage language development. In sum, this study highlights a novel approach for studying the brain bases of bilingual language development and reveals that young bilinguals can engage in sensitive lexical processing of their heritage language even within a broader majority language context.

## Supplementary Material

Appendix A

## Figures and Tables

**Table 1:** Participants’ behavioral assessment scores in both languages.

	Spanish	English
Morphosyntax	15 (± 6.0)	29 (± 4.6)
Listening Comprehension	33 (± 7.7)	33 (± 7.1)
Production	26 (± 10)	29 (± 8.4)
Non-word Reading	32 (± 17)	31 (± 12)
Sentence Reading Fluency	25 (± 13)	40 (± 16)
Input (hours per week)	51 (± 16)	73 (± 19)
Output (hours per week)	46 (± 20)	73 (± 22)

*Note*. Table shows mean ± standard deviation. Assessment scores show raw values. Morphosyntax scores are from BESA-ME assessments. Listening comprehension scores and production scores are from TNL assessments. Non-word reading scores are from IDEL and TOWRE. Input and output values were derived from the BIOS-Home measure and reflect hours per week in that language. All values had three or fewer missing cases, except for Spanish Listening Comprehension (*n* = 5), Spanish Production (*n* = 6), and Spanish Sentence Reading Fluency (*n* = 8).

## Data Availability

Data and analysis scripts are available on OSF: https://osf.io/fxzpk/overview

## Footnotes

1 At a reviewer’s suggestion, we re-ran analyses with these five language/reading disorder participants excluded and found a similar pattern of results. The analyses and versions of Fig. 1 & 2 with these five participants excluded are available on OSF.

2 At a reviewer’s suggestion, we re-ran analyses excluding the five participants with below 60% accuracy and again excluding the seven participants with below 80% accuracy. In both cases, we found a similar pattern of results (with slight differences for the exploratory whole brain-proficiency results). The analyses and versions of Figures 1 & 2 with low-accuracy participants excluded are available on OSF.

## Appendix A. Supplementary data

Supplementary data to this article can be found online at https://doi.org/10.1016/j.bandl.2026.105833.
