# Supplementary material for: Neural tracking of surprisal in Spanish-English bilingual children during naturalistic heritage language listening
Source: Brain Lang. Author manuscript; Available in PMC 2026 Sep 21. (PMC13592032; doi:10.1016/j.bandl.2026.105833)
Supplement: Supplementary file 1 [file NIHMS2208959-supplement-Appendix_A.docx]

# Supplementary Materials

**Supplementary Figure 1**

*Probeset*

*Note.* fNIRS probeset, with sources shown with red squares and detectors with blue circles. There are fewer channels in the right hemisphere, as one detector is used to form short channels. This figure is taken from Eggleston, 2025 “Morphological Awareness and Word Reading in Developing Readers: A Brain-Behavior Investigation,” which also includes a table (Appendix Table B.1.) that maps our channels onto MNI space.

**Supplementary Table 1**

*Main Model Results*

| **channel** | **regressor** | **beta** | **se** | **tstat** | **p** | **q** |
| --- | --- | --- | --- | --- | --- | --- |
| left 1 | word_rate | 2.101518 | 3.488167 | 0.602471 | 0.547384 | 0.778502 |
| left 1 | surprisal | -0.03122 | 0.165101 | -0.1891 | 0.850162 | 0.917029 |
| left 1 | word_freq | -0.89368 | 0.963185 | -0.92784 | 0.354347 | 0.654179 |
| left 2 | word_rate | 2.571467 | 2.634255 | 0.976165 | 0.329887 | 0.639771 |
| left 2 | surprisal | 0.100106 | 0.136114 | 0.735453 | 0.462724 | 0.725916 |
| left 2 | word_freq | -1.59042 | 0.726554 | -2.189 | 0.029483 | 0.187079 |
| left 3 | word_rate | 2.555957 | 3.659725 | 0.698402 | 0.485548 | 0.745802 |
| left 3 | surprisal | 0.216444 | 0.172653 | 1.253638 | 0.211095 | 0.500739 |
| left 3 | word_freq | -1.46551 | 1.005816 | -1.45703 | 0.14631 | 0.445897 |
| left 4 | word_rate | 6.285059 | 3.588004 | 1.751687 | 0.081002 | 0.311049 |
| left 4 | surprisal | -0.01553 | 0.172131 | -0.09024 | 0.928162 | 0.963373 |
| left 4 | word_freq | -1.51577 | 0.99259 | -1.52709 | 0.127951 | 0.409442 |
| left 5 | word_rate | 15.10477 | 3.558686 | 4.24448 | 3.05E-05 | 0.001463 |
| left 5 | surprisal | 0.366284 | 0.168076 | 2.179276 | 0.030206 | 0.187079 |
| left 5 | word_freq | -3.86151 | 0.979029 | -3.94422 | 0.000103 | 0.003953 |
| left 6 | word_rate | 5.681999 | 4.274496 | 1.329279 | 0.184917 | 0.493612 |
| left 6 | surprisal | -0.29184 | 0.202145 | -1.44371 | 0.150019 | 0.450057 |
| left 6 | word_freq | -1.06129 | 1.182632 | -0.8974 | 0.370332 | 0.663791 |
| left 7 | word_rate | 16.82062 | 3.497502 | 4.809325 | 2.56E-06 | 0.000236 |
| left 7 | surprisal | 0.799118 | 0.165033 | 4.842177 | 2.20E-06 | 0.000236 |
| left 7 | word_freq | -3.74525 | 0.964712 | -3.88225 | 0.000131 | 0.004198 |
| left 8 | word_rate | 2.283999 | 3.737572 | 0.611091 | 0.541671 | 0.778502 |
| left 8 | surprisal | -0.38146 | 0.177539 | -2.14857 | 0.032588 | 0.193896 |
| left 8 | word_freq | -0.33738 | 1.030554 | -0.32737 | 0.743647 | 0.874105 |
| left 9 | word_rate | 3.077408 | 2.880194 | 1.068472 | 0.286295 | 0.62449 |
| left 9 | surprisal | 0.506686 | 0.136914 | 3.700759 | 0.000262 | 0.007191 |
| left 9 | word_freq | -1.9173 | 0.793282 | -2.41692 | 0.016339 | 0.142592 |
| left 10 | word_rate | 1.106488 | 5.020741 | 0.220383 | 0.825745 | 0.900767 |
| left 10 | surprisal | -0.50251 | 0.240338 | -2.09086 | 0.037508 | 0.211811 |
| left 10 | word_freq | -0.34253 | 1.38328 | -0.24762 | 0.804623 | 0.884028 |
| left 11 | word_rate | -1.53192 | 3.86703 | -0.39615 | 0.692318 | 0.872206 |
| left 11 | surprisal | 0.193024 | 0.183086 | 1.05428 | 0.29273 | 0.62449 |
| left 11 | word_freq | 0.102468 | 1.061582 | 0.096524 | 0.923179 | 0.963373 |
| left 12 | word_rate | 5.77589 | 4.226647 | 1.366542 | 0.172945 | 0.478848 |
| left 12 | surprisal | -0.06342 | 0.202226 | -0.31359 | 0.754082 | 0.874105 |
| left 12 | word_freq | -1.45637 | 1.165462 | -1.24961 | 0.212562 | 0.500739 |
| left 13 | word_rate | -4.48529 | 4.395791 | -1.02036 | 0.308502 | 0.631427 |
| left 13 | surprisal | 0.093406 | 0.207358 | 0.450455 | 0.652756 | 0.85258 |
| left 13 | word_freq | 1.660539 | 1.208061 | 1.37455 | 0.17045 | 0.478848 |
| left 14 | word_rate | -3.55445 | 3.511937 | -1.01211 | 0.312425 | 0.631427 |
| left 14 | surprisal | 0.00512 | 0.172023 | 0.029764 | 0.976278 | 0.98139 |
| left 14 | word_freq | 1.217943 | 0.973543 | 1.251041 | 0.21204 | 0.500739 |
| left 15 | word_rate | 1.954609 | 2.629076 | 0.743459 | 0.457873 | 0.725916 |
| left 15 | surprisal | -0.04736 | 0.128256 | -0.36927 | 0.712222 | 0.872206 |
| left 15 | word_freq | -0.95553 | 0.728175 | -1.31222 | 0.190598 | 0.496369 |
| left 16 | word_rate | -1.48246 | 4.128366 | -0.35909 | 0.719817 | 0.872584 |
| left 16 | surprisal | 0.049047 | 0.197682 | 0.24811 | 0.804244 | 0.884028 |
| left 16 | word_freq | 0.139048 | 1.14276 | 0.121677 | 0.903248 | 0.952877 |
| left 17 | word_rate | 2.145404 | 4.071278 | 0.526961 | 0.598668 | 0.826938 |
| left 17 | surprisal | -0.21162 | 0.191615 | -1.10438 | 0.270446 | 0.596846 |
| left 17 | word_freq | -0.36135 | 1.126006 | -0.32092 | 0.748531 | 0.874105 |
| right 1 | word_rate | -2.90348 | 3.30377 | -0.87884 | 0.380297 | 0.663791 |
| right 1 | surprisal | 0.342681 | 0.156388 | 2.191225 | 0.02932 | 0.187079 |
| right 1 | word_freq | -0.50073 | 0.911339 | -0.54944 | 0.583171 | 0.811369 |
| right 2 | word_rate | 1.37014 | 2.789114 | 0.491246 | 0.623666 | 0.849247 |
| right 2 | surprisal | 0.10901 | 0.133953 | 0.813798 | 0.416503 | 0.695378 |
| right 2 | word_freq | -0.28444 | 0.773048 | -0.36795 | 0.71321 | 0.872206 |
| right 3 | word_rate | 1.276376 | 3.130067 | 0.407779 | 0.68377 | 0.871156 |
| right 3 | surprisal | 0.154464 | 0.149126 | 1.035796 | 0.301256 | 0.631427 |
| right 3 | word_freq | -1.12406 | 0.857986 | -1.31012 | 0.191309 | 0.496369 |
| right 4 | word_rate | -3.65557 | 3.863136 | -0.94627 | 0.344886 | 0.642894 |
| right 4 | surprisal | -0.03941 | 0.183788 | -0.21441 | 0.830394 | 0.900767 |
| right 4 | word_freq | 0.439 | 1.065213 | 0.412125 | 0.680586 | 0.871156 |
| right 5 | word_rate | 12.64797 | 3.897569 | 3.245092 | 0.001327 | 0.025486 |
| right 5 | surprisal | 0.342787 | 0.185987 | 1.843066 | 0.066453 | 0.278259 |
| right 5 | word_freq | -3.06843 | 1.065668 | -2.87935 | 0.004315 | 0.063735 |
| right 6 | word_rate | 2.164298 | 4.747965 | 0.455837 | 0.648886 | 0.85258 |
| right 6 | surprisal | 0.350552 | 0.223722 | 1.56691 | 0.118347 | 0.394593 |
| right 6 | word_freq | -1.27348 | 1.308668 | -0.97311 | 0.331399 | 0.639771 |
| right 7 | word_rate | 6.167249 | 3.309751 | 1.863357 | 0.063535 | 0.278259 |
| right 7 | surprisal | 0.742251 | 0.156929 | 4.729867 | 3.68E-06 | 0.000236 |
| right 7 | word_freq | -1.54302 | 0.911176 | -1.69344 | 0.091565 | 0.344714 |
| right 8 | word_rate | -0.12102 | 3.530112 | -0.03428 | 0.972678 | 0.98139 |
| right 8 | surprisal | 0.244948 | 0.172499 | 1.419994 | 0.156803 | 0.463172 |
| right 8 | word_freq | -0.62114 | 0.976472 | -0.63611 | 0.525263 | 0.764019 |
| right 9 | word_rate | -8.16869 | 3.360923 | -2.43049 | 0.015753 | 0.142592 |
| right 9 | surprisal | 0.286515 | 0.159409 | 1.797351 | 0.073436 | 0.293743 |
| right 9 | word_freq | 1.138926 | 0.923019 | 1.233914 | 0.218345 | 0.502924 |
| right 10 | word_rate | -11.5206 | 5.972669 | -1.92888 | 0.05483 | 0.269934 |
| right 10 | surprisal | -0.22374 | 0.280637 | -0.79724 | 0.426037 | 0.697339 |
| right 10 | word_freq | 2.766233 | 1.644191 | 1.682428 | 0.093682 | 0.345902 |
| right 11 | word_rate | -3.08852 | 3.405847 | -0.90683 | 0.365334 | 0.661737 |
| right 11 | surprisal | 0.027696 | 0.161996 | 0.170968 | 0.864381 | 0.927158 |
| right 11 | word_freq | 0.991465 | 0.936323 | 1.058892 | 0.290628 | 0.62449 |
| right 13 | word_rate | -5.49961 | 3.942822 | -1.39484 | 0.16425 | 0.477818 |
| right 13 | surprisal | 0.309912 | 0.186623 | 1.66063 | 0.097989 | 0.348404 |
| right 13 | word_freq | 2.488039 | 1.081844 | 2.299814 | 0.022248 | 0.15821 |
| right 15 | word_rate | 4.210289 | 3.263103 | 1.290272 | 0.198098 | 0.500739 |
| right 15 | surprisal | -0.33573 | 0.156922 | -2.13946 | 0.033326 | 0.193896 |
| right 15 | word_freq | -1.10352 | 0.902725 | -1.22243 | 0.222648 | 0.502924 |
| right 16 | word_rate | -3.33905 | 4.02104 | -0.83039 | 0.407075 | 0.689883 |
| right 16 | surprisal | -0.12306 | 0.191965 | -0.64106 | 0.522048 | 0.764019 |
| right 16 | word_freq | 1.015784 | 1.1151 | 0.910935 | 0.36317 | 0.661737 |
| right 17 | word_rate | 2.079436 | 4.384855 | 0.474231 | 0.635731 | 0.85258 |
| right 17 | surprisal | -0.38138 | 0.20709 | -1.84161 | 0.066666 | 0.278259 |
| right 17 | word_freq | 0.012751 | 1.213382 | 0.010508 | 0.991624 | 0.991624 |

*Note.* Table shows the results of the main model, which was run using the NIRS brain AnalyzIR toolbox. Figure 1 in the paper visualizes these results. Only HbO results are reported. See Supplementary Figure 1 for a visualization of channel locations in our probeset.

**Supplementary Figure 2**

*ROI Brain-Proficiency Relationships*

*Note.* Median values and 95% credibility interval for the estimated correlation between each proficiency measure and the STG ROI effect of surprisal. Y-axis represents the four separate Bayesian models that were run.

**Supplementary Table 2**

*ROI Brain-Proficiency Model: English Listening*

|  | **Estimate** | **Est.Error** | **l-95% CI** | **u-95% CI** | **Rhat** | **Bulk_ESS** | **Tail_ESS** |
| --- | --- | --- | --- | --- | --- | --- | --- |
| Intercept | 1.72 | 2 | -2.23 | 5.62 | 1 | 5288 | 2904 |
| English listening | 0.05 | 0.05 | -0.04 | 0.15 | 1 | 3053 | 2491 |
| age | -0.3 | 0.24 | -0.76 | 0.17 | 1 | 3331 | 2579 |
|  |  |  |  |  |  |  |  |

**Supplementary Table 3**

*ROI Brain-Proficiency Model: Spanish Listening*

|  | **Estimate** | **Est.Error** | **l-95% CI** | **u-95% CI** | | **Rhat** | **Bulk_ESS** | **Tail_ESS** |
| --- | --- | --- | --- | --- | --- | --- | --- | --- |
| Intercept | 3.84 | 2.12 | -0.39 | 8.16 | 1 | | 3881 | 2928 |
| Spanish listening | -0.06 | 0.04 | -0.15 | 0.01 | 1 | | 3521 | 2695 |
| age | -0.1 | 0.21 | -0.53 | 0.32 | 1 | | 3480 | 2810 |

**Supplementary Table 4**

*ROI Brain-Proficiency Model: English Reading*

|  | **Estimate** | **Est.Error** | **l-95% CI** | **u-95% CI** | | **Rhat** | **Bulk_ESS** | **Tail_ESS** |
| --- | --- | --- | --- | --- | --- | --- | --- | --- |
| Intercept | 2.4 | 2.07 | -1.62 | 6.42 | 1 | | 2796 | 2506 |
| English reading | 0 | 0.02 | -0.04 | 0.04 | 1 | | 2339 | 2463 |
| age | -0.18 | 0.28 | -0.74 | 0.35 | 1 | | 2223 | 2382 |

**Supplementary Table 5**

*ROI Brain-Proficiency Model: Spanish Reading*

|  | **Estimate** | **Est.Error** | **l-95% CI** | **u-95% CI** | **Rhat** | **Bulk_ESS** | **Tail_ESS** |
| --- | --- | --- | --- | --- | --- | --- | --- |
| Intercept | 2.02 | 1.91 | -1.65 | 5.67 | 1 | 3897 | 2934 |
| Spanish reading | -0.02 | 0.02 | -0.07 | 0.02 | 1 | 3304 | 2920 |
| age | -0.08 | 0.23 | -0.53 | 0.38 | 1 | 3169 | 2602 |

**Supplementary Figure 3**

*Proficiency–Input Correlation Matrix*

*Note.* Shows visualization of correlations between age, input, and proficiency measures. Listening comprehension was measured with TNL assessments and reading comprehension with sentence reading fluency subtest. Assessment scores are raw scores. Input was measured in hours per week. Color indicates r value. (*p<0.05, **p<0.01, ***p<0.001)
